# Protective Effect of Naringin in L-arginine-induced Acute Pancreatitis in Wistar Rats

**DOI:** 10.31661/gmj.v13i.3354

**Published:** 2024-09-06

**Authors:** Navid Moznebi Esfahani, Seyed Alireza Salimi Tabatabaee, Fatemeh Karamali, Seyed Abbas Mirmalek, Shima Shafagh, Nushin Moussavi

**Affiliations:** ^1^ Trauma Research Center, Kashan University of Medical Sciences, Kashan, Iran; ^2^ Department of Cardiovascular Medicine, Kashan University of Medical Sciences, Kashan, Iran; ^3^ Department of Surgery, Tehran Medical Sciences, Islamic Azad University, Tehran, Iran

**Keywords:** Naringin, L-arginine Pancreatitis, Oxidative Stress, Anti-inflammation

## Abstract

Background: Acute pancreatitis, a non-infectious inflammatory disorder of the pancreas, is not only the most common cause of hospitalization among gastrointestinal diseases in many countries but up to 20% of patients may experience morbidity and mortality. Naringin is a common flavonoid that is found in many fruits such as oranges and tomatoes, and evidence revealed its use in the prevention and treatment of many diseases due to its antioxidant and anti-inflammatory effects. Hence, this study was conducted to investigate the anti-inflammatory and antioxidant effects of naringin in the pancreatitis model in rats. Materials and Methods: In this experimental study, sixty male Sprague-Dawley rats were divided into 4 equal groups. In the control group, normal saline was injected intraperitoneally (IP). In the sham and experimental groups, pancreatitis was induced with a dose of 3.2 g/kg body weight of L-arginine IP, twice with a time interval of one hour. Rats of low dose (E-L) and high dose (E-H) groups were treated with 200 and 500 mg/kg of naringin IP, 30 minutes before L-arginine administration, respectively. Serum lipase and amylase along with pancreatic IL-10, IL-1β, and TNF-α were measured. Also, to evaluate oxidative stress, pancreatic superoxide dismutase (SOD), glutathione (GSH), malondialdehyde (MDA), and myeloperoxidase (MPO) were evaluated. In addition, the histopathological study was performed with morphological examination. Results: Sham rats exhibited increased levels of amylase and lipases compared to controls. Naringin administration significantly reduced these levels in the experimental groups. In addition, naringin decreased MDA and MPO levels and increased SOD and GSH activities in the E-L and E-H groups. TNF-α and IL-1β levels were higher in the sham group but reduced with naringin treatment. Naringin also increased IL-10 levels in a dose-dependent manner. Histopathological analysis showed that naringin reduced tissue damage severity in a dose-dependent manner. Conclusion: Based on the results obtained in the study, naringin administration effectively reduced pancreas enzyme activity, and increased antioxidant enzyme activities in rats with induced pancreatitis. Naringin also exhibited anti-inflammatory effects by decreasing TNF-α and IL-1β levels while increasing IL-10 levels in a dose-dependent manner. Moreover, the histopathological analysis demonstrated that naringin had protective effects against tissue damage caused by pancreatitis, showing a dose-dependent reduction in the severity of edema, inflammation, and necrosis. These findings suggest that naringin holds promise as a potential therapeutic agent for managing pancreatitis-related complications.

## Introduction

Acute pancreatitis stands as a major medical challenge characterized by the rapid onset of inflammation within the pancreas, often leading to severe complications and significant morbidity and mortality rates [1][2]. Indeed, pancreatitis is typically marked by the inappropriate activation of pancreatic enzymes, resulting in the autodigestion of pancreatic tissue and the release of pro-inflammatory mediators [3]. Naringin, a flavonoid compound abundantly found in citrus fruits such as grapefruits and oranges, has received attention for its diverse pharmacological properties, including anti-inflammatory, antioxidant, and cytoprotective effects [4][5]. Previous studies have indicated naringin’s potential to ameliorate oxidative stress [6], modulate inflammatory pathways [7], and preserve cellular integrity [8] in various disease conditions. Regarding the role of oxidative stress, i.e., reactive oxygen species (ROS) as one the main factors in the pathophysiology of pancreatitis, this study aimed to evaluate the anti-inflammatory and anti-oxidative effects of naringin on the acute pancreatitis model in Wistar rats.

## Materials and Methods

Study Design And Groups

Sixty male Sprague-Dawley rats, weighing 180-200 g, were obtained from the Pasteur Institute, Tehran, Iran. Rats were housed individually in cages on standard condition (a 12:12 h light-dark cycle at 23°C) and free access to pellet diet and water ad libitum for a week prior to experiments. Rats were randomly divided into four groups (n=15 per group) as follows:

-Control group: received intraperitoneal (IP) injections of normal saline.

-Sham group: for induced acute pancreatitis in rats, 3.2 g/kg bodyweight (b.w) L-arginine (Sigma-Aldrich, Germany) was injected IP, twice at an interval of one hour [9]

-Experimental groups: Rats in E-L and E-H groups were treated as low- and high-dose groups with a single dose of 200 and 500 mg/kg b.w naringin (Sigma-Aldrich, Germany) IP, 30 minutes prior to L-arginine administration [10], respectively.

Samples Collection

All rats were sacrificed with an overdose of pentobarbital 24 hours after the last injection of L-arginine. Blood samples were obtained by direct intracardiac puncture and stored at -70°C for biochemical analysis. The pancreas (five rats per group) was quickly removed and fixed in formaldehyde (10%) for histological examination.

Serum Amylase and Lipase Levels Determination

Blood samples were centrifuged at 15,000 rpm under 4°C and the plasma was separated by using sterile pipettes. Serum lipase and amylase activity were evaluated with a spectrophotometric technique by the Olympus AU-2700 autoanalyzer (Olympus, Hamburg, Germany) using commercial kits (MAN Company, Tehran, Iran), and results were expressed as U/I.

Measurement of Serum Inflammatory Cytokines

Serum IL-10, IL-1β, and TNF-α levels were measured using an enzyme-linked immunosorbent assay (ELISA) based Mirmalek et al. study [9]. Briefly, the blood sample of each group was centrifuged at 3500 r min−1 for 15 min. The supernatant was obtained for the analysis of cytokines. These cytokines were measured with ELISA kits (Boster Biological Technology, Wuhan, China) according to the manufacturer’s protocol. The ELISA microplate was read using an ELISA reader (Dynatech Laboratories, USA) with an absorbance maximum at 450 nm. The cytokine levels were calculated after plotting the standard curves and expressed as pg/mL.

ROS Detection

To evaluate oxidative stress status, five rats from each group were randomly selected and pancreatic tissues were removed, frozen in liquid nitrogen, and stored at -70°C until being assayed. Protein estimation was done by the method of Lowry et al. [11]. Also, proper commercial kits and previous described methods by Mirmalek et al. [9] were applied for the determination of pancreatic oxidative and anti-oxidative contents as follows:

1. SOD Activity

The activity of SOD was measured using assay kit (Sigma, Germany) based on the manufacturer’s instructions. Briefly, this kit uses a tetrazolium salt for the detection of superoxide anions generated by xanthine oxidase and hypoxanthine. These superoxide radicals oxidize hydroxylamine and lead to the formation of nitrite, which reacts with naphthalene diamine and sulfanilic acid to produce a colored product. SOD in the sample reduces the overall superoxide anion concentration, thereby lowering the colorimetric signal and absorbance at 550 nm. One unit (U) of SOD was defined as the amount of enzyme needed to produce 50% dismutation of superoxide radical. The activity of SOD was expressed as U/mg of protein.

2. GSH Content

The GSH content was measured using the 5,5′-dithiobis (2-nitrobenzoic acid)-oxidized GSH (DTNB-GSSG) reductase recycling assay (Sigma, Germany) for total glutathione (GSH + GSSG). Briefly, tissues were lysed by 200 μL of lysis buffer (50 mM Tris-HCl, 1 mM EGTA, and 1% Triton X-257 100). The tissue lysate was deproteinized with the same volume of 10% 5-sulfosalicylic acid. After centrifugation at 5000 g for 5 min at 4°C, the supernatant was divided into two samples for GSH and GSSG measures. The amount of total GSH was determined by the formation of 5-thio-2-nitrobenzoic acid converted from DTNB. GSSG was measured by the DTNB-GSSG reductase recycling assay after treating GSH with 2-vinylpyridine for one hour at room temperature. Total glutathione and GSSG levels were defined as the change in optical density at 405 nm for 5 min at room temperature. The results were expressed as μmol/g.

3. MDA Content

The MDA content was determined using the thiobarbituric acid (TBA) test by a commercial kit (Sigma, Germany). In brief, samples were homogenized in 10 mL of TCA (7.5%)-EDTA (0.1%) solution. This sample was shaken continuously for 30 min with a mechanical shaker and then filtered. Exactly 5 mL of filtrate was added to 5 mL of TBA (2.88 g/L) solution in a 25 mL colorimetrical tube and heated in a water bath (90°C) for 40 min for pink color development. The tube was first cooled for one hour and was then centrifuged for 5 min at 3000 g. The supernatant fluid was added to 5 mL of chloroform in another tube and then shaken. This mixed solution was allowed to stand for at least one hour. The absorbance was measured at 532 nm using a spectrophotometer (UV-2550, Shimadzu, Kyoto, Japan). The results were expressed as nmol/g of protein.

4. MPO Activity

The MPO activity of pancreatic was determined as described by Bradley et al. [12]. Tissue samples were homogenized in 50 mM potassium phosphate buffer (PB, pH 6.0) and centrifuged at 41, 400 g (10 min); pellets were suspended in 50 mM PB containing 0.5% hexadecyltrimethylammonium bromide (HETAB). After three freeze and thaw cycles, with sonication between cycles, the samples were centrifuged at 41, 400 g for 10 min. Aliquots (0.3 mL) were added to 2.3 mL of the reaction mixture containing 50 mM PB, o-dianisidine, and 20 mM H2O2 solution. One unit of enzyme activity was defined as the amount of MPO present that caused a change in absorbance measured at 460 nm for 3 min. MPO activity was expressed as U/g protein.

Histopathological Evaluations

Histopathological changes in the pancreas tissues were evaluated according to a scoring system as previously described by Schmidt et al. [13]. Hence, paraffin-embedded pancreas tissues were sectioned (5 μm) and stained with hematoxylin and eosin. Then, semiquantitative assessment of edema, inflammatory cell infiltrate, and acinar necrosis were performed as follows:

1. Edema: 0 = absent, 1 = focally increased between lobules, 2 = diffusely increased between lobules, and 3 = acini disrupted and separated;

2. Inflammatory cell infiltration: 0 = absent, 1 = rare or around ductal margins, 2 = in the parenchyma (<50% of the lobules), and 3 = in the parenchyma (>50% of the lobules);

3. Necrosis: 0 = absent, 1 = architectural changes, 2 = pycnotic nuclei, 3 = focal necrosis (<10% of the parenchyma), and 4 = diffuse parenchymal necrosis (>10% of the parenchyma). Finally, the severity of acute pancreatitis was graded by the sum of the scores of all three sections as mentioned.

Ethical Issues

All procedures were performed according to the Guide for the Care and Use of Laboratory Animals (NIH publication number 86-23, 1985 edition) and approved by the Research Ethics Committees of Laboratory Animals of Kashan University of Medical Sciences and Health Services (code: IR.KAUMS.AEC.1402.009).

Statistical Analysis

All results were expressed as mean ± standard deviations (SD) and were analyzed using GraphPad Prism software (version 6.01, GraphPad, La Jolla, CA, USA). Also, one-way analysis of variance (ANOVA) followed by Tukey’s multiple comparison tests, as well as, the Mann-Whitney test for nonparametric data were applied. The significance level was set at P=0.05.

## Results

**Figure-1:**
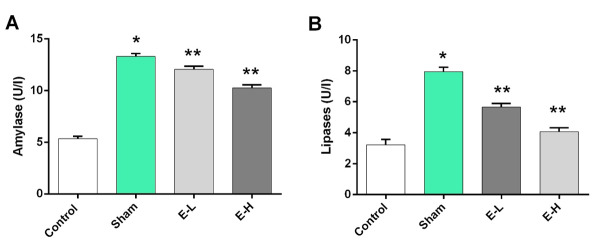
Figure 1. Serum amylase (A) and lipase (B) of rats in all the groups. *P<0.001 vs. control, **P<0.001 vs. sham.

Naringin Could Armillarioid Pancreas Enzymes Activity

To measure pancreas enzyme activity, amylase and lipase levels were determined in the serum of all rats. Rats in the sham group showed significant elevation of amylase (13.31±0.25 U/I) and lipases (7.94±0.28 U/I) levels in comparison with the control group (5.33±0.23 and 3.22±0.35, respectively, P<0.001, Figure-1). While pancreas enzymes were increased in experimental groups vs. control group (Figure-1); however, these contents were markedly (P<0.001) decreased in E-L and E-H groups after naringin administrants in comparison with sham group. Also, there were no any significant differences between the E-L and E-H groups in terms of pancreas enzyme activity (P=0.063).

ROS Formation Reduced By Naringin Administration Via Antioxidative Component

As shown in Table-1, MPO and MDA levels in rats of the sham group were higher than those of the control group, while SOD and GSH levels were significantly lower than those of the control group. Also, MDA and MPO levels were significantly increased in the pancreatitis rats, whereas the activities of antioxidant enzymes, such as SOD and GSH, were decreased (Table-1). However, treatment of rats with naringin effectively decreased MDA and MPO levels and increased antioxidant enzyme activities in E-L and E-H groups (Table-1).

Anti-inflammatory Effects of Naringin Via Reduction of TNF-α and IL-1β

**Figure-2:**
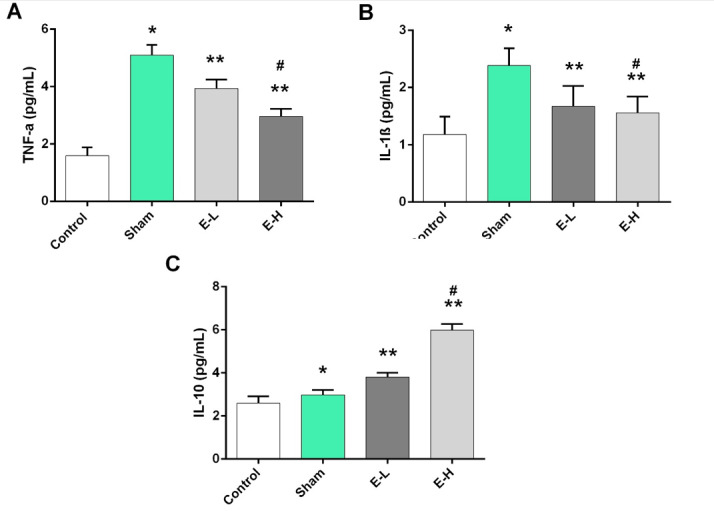
Figure 2. Serum cytokines contents of rats. The proinflammatory TNF-α (A) and IL-1β (B) significantly lowered in E-L and E-H groups compared with the sham group. However, treatment rats with naringin markedly increased anti-inflammatory IL-10 levels (C) compared to control and sham groups. *P<0.001 vs. control, **P<0.001 vs. sham, #P< 0.001 vs. E-H.

Our study revealed that TNF-α and IL-1β levels in the control group were significantly lower than in the sham group (Figure-2A and B). However, in terms of TNF-α and IL-1β levels, significant differences were observed between sham and treatment groups that indicated the anti-inflammatory effects of naringin. Regarding Figure-2, compared to the sham group, treatment of rats with low- and high-dose naringin significantly increased IL-10 levels in experimental groups (P=0.011). Also, the finding indicated that the anti-inflammatory properties of naringin elevated by increasing the IL-10 level in a dose manner (3.81±0.19 vs. 5.97±0.29 pg/mL).

Treatment Rats With Naringin Could Markedly Attenuate Severity Of Acute Pancreatitis

Histopathological examination revealed that the tissue damages caused by L-arginine were significantly higher in animals that were subjected to pancreatitis than in the control group. Also, in terms of the severity of edema, inflammation, and necrosis, the sham group was significantly higher than the treatment groups. In other words, treatment with naringin markedly reduced the severity of tissue damage, which indicates its protective effects in a dose-dependent manner.

## Discussion

**Table:** Table1. Oxidative Stress Status Of Rats In All Groups

**Parameters**	**Control** **(mean**±SD)	**Sham** **(mean**±SD)	**E-L** **(mean**±SD)	**E-H** **(mean**±SD)
**MDA (nmol/g)**	1.97±0.33	4.6±0.23 ^a^	3.23±0.3 ^a,b^	2.69±0.31 ^a,b,c^
**MPO (U/g)**	0.54±0.24	0.88±0.33 ^a^	0.77±0.34 ^a,b^	0.56±0.24 ^a,b,c^
**SOD (U/mL)**	4.85±0.03	3.3±0.22 ^a^	10.14±0.37 ^a,b^	11.96±0.21 ^a,b,c^
**GSH (µmol/g)**	0.57±0.33	2.89±0.3 ^a^	6.15±0.29 ^a,b^	9.56±0.27 ^a,b,c^

aP<0.001 vs. control, bP<0.001 vs. sham, cP<0.005 vs. E-L

In the present study, the protective role of naringin against acute pancreatitis caused by L-arginine in a rat model. The results showed that treating rats with naringin could significantly reduce the elevation of amylase and lipase following damage to the pancreas. Also, the findings of the present study showed that naringin exerts its protective effects through its anti-inflammatory properties by reducing inflammatory cytokines (i.e., TNF-α and IL-1β) and simultaneously increasing the amount of anti-inflammatory cytokine, i.e., IL-10. On the other hand, by reducing oxidative enzymes in contrast to enhancing antioxidant properties by increasing SOD and GSH levels, naringin shows its protective effects against pancreatic damage.

The current study evaluates pancreas enzyme activities, particularly the assessment of amylase and lipase levels in the serum of rats, and elucidates the impact of naringin on pancreatic function in the context of acute pancreatitis. The significant elevation of amylase and lipase levels in the sham group compared to the control group was indicative of pancreas damage, consistent with previous studies demonstrating the correlation between elevated enzyme levels and pancreatic injury [14]. This finding emphasizes the reliable nature of enzyme assays as biomarkers for pancreatic health and the pathological changes associated with pancreatitis.

The observed increase in pancreas enzyme activity in the experimental groups compared to the control group suggests a model of induced pancreatitis successfully in the current study. These findings were in line with previous research by Yang et al. [15] and Su et al. [16], indicating that experimental models involving enzyme imbalances can effectively mimic pathological conditions seen in pancreatitis. The subsequent reduction in enzyme levels in both the E-L and E-H groups following naringin administration highlights the therapeutic potential of naringin in mitigating pancreas enzyme activity and preserving pancreatic function. The current literature supports the beneficial effects of naringin in ameliorating enzyme imbalances and reducing pancreatic damage in various disease models [17][18].

Moreover, the lack of significant differences between the E-L and E-H groups in terms of pancreas enzyme activity post-naringin treatment showed the need for optimal dosage of naringin for therapeutic efficacy. Indeed, the findings of Chattopadhyay et al. [19] and Alam et al. [20] studies suggest that the dose-dependent effects of naringin may vary across different experimental contexts, warranting further investigations to elucidate the optimal dosage range for maximizing its therapeutic benefits against pancreatitis.

Regarding previous studies [21][22], naringin's ability to modulate inflammatory responses and oxidative stress in different disease models revealed its broad spectrum of therapeutic actions. Furthermore, our study revealed pathways through which naringin exerts its protective effects in acute pancreatitis. The downregulation of proinflammatory cytokines observed in the experimental groups that received naringin indicates a potential mechanism underlying its anti-inflammatory actions. Indeed, naringin may attenuate the inflammatory cascade and reduce tissue injury, as evidenced by the decreased levels of pro-inflammatory cytokines and increased IL-10 in our study.

Also, our data highlights that in the sham group, MPO and MDA levels were higher while SOD and GSH levels were lower compared to the control group. Moreover, pancreatitis rats exhibited a significant increase in MDA and MPO levels alongside decreased antioxidant enzyme activities, such as SOD and GSH. However, the administration of naringin to rats led to a notable decrease in MDA and MPO levels while simultaneously boosting the activities of antioxidant enzymes in the E-L and E-H groups. Current literature and previous research support these findings [23][24][25]. Oxidative stress, characterized by an imbalance between the production of ROS and the antioxidant defense system, has been implicated in various diseases, including pancreatitis [26]. Studies have shown that increased MDA and MPO levels are indicative of lipid peroxidation and neutrophil infiltration, respectively, which contribute to tissue damage in pancreatitis [27]. On the other hand, reduced SOD and GSH levels signify compromised antioxidant defense mechanisms, making cells more susceptible to oxidative damage [28].

The beneficial effects of naringin in ameliorating oxidative stress and bolstering antioxidant defenses align with previous evidence [29][30] on the antioxidant properties of flavonoids. Naringin, a flavonoid present in some fruits, has been reported to possess potent antioxidant [22] and anti-inflammatory [30] properties. It can scavenge ROS, inhibit lipid peroxidation, and enhance the activity of antioxidant enzymes like SOD and GSH [31]. By modulating these pathways, naringin can mitigate oxidative damage and inflammation, thereby exerting protective effects in various disease models, including pancreatitis [32].

While the current study provides valuable insights into the protective effects of naringin in experimental acute pancreatitis, several limitations should be considered. The use of a rat model may not fully reflect the complexity of human pancreatitis, and further research in translational models is essential to validate the efficacy of naringin in clinical settings. Moreover, investigating the long-term effects, potential side effects, and interaction profiles of naringin with other medications should be crucial for its safe and effective use in clinical practice. Future research should also elucidate the precise molecular mechanisms underlying naringin's protective effects in pancreatitis and identify specific targets for therapeutic intervention to advance its clinical development as a novel treatment option for acute pancreatitis.

## Conclusion

Our study indicates that naringin, as a natural compound with multiple pharmacological actions could protect from damage to pancreatic via different pathways, including increased anti-inflammatory cytokines and reduction of ROS formation using decrees MDA and MPO levels while SOD and GSH were elevated.

## Conflict of Interest

There are no any conflicts of interest.
